# Therapeutic Effects of Lurbinectedin in PARP Inhibitor-Resistant High-Grade Serous Ovarian Cancer Using Patient-Derived Cell Lines and Organoid Models

**DOI:** 10.3390/ijms26188866

**Published:** 2025-09-11

**Authors:** Akshi Srivastava, Christine Unterweger, Sarah Guttmann, Andrea Wolf, Leonhard Müllauer, Martin Schepelmann, Dan Cacsire Castillo-Tong, Thomas Bartl

**Affiliations:** 1 Translational Gynecology Group, Department of Obstetrics and Gynecology, Medical University of Vienna, 1090 Vienna, Austriachristine.unterweger@meduniwien.ac.at (C.U.);; 2 Department of Pathology, Medical University of Vienna, 1090 Vienna, Austria; 3 Institute of Pathophysiology and Allergy Research, Center of Pathophysiology, Infectiology and Immunology, Medical University of Vienna, 1090 Vienna, Austria; martin.schepelmann@meduniwien.ac.at

**Keywords:** PARPi resistance, high-grade serous ovarian cancer, patient-derived cell lines and organoids, lurbinectedin, preclinical models

## Abstract

High-grade serous ovarian cancer (HGSOC) is an aggressive malignancy which is often treated with platinum-based chemotherapy and PARP inhibitors (PARPi). However, PARPi resistance remains a major clinical challenge, necessitating alternative therapeutic strategies. In this study, we establish the first known patient-derived organoid models directly from PARPi-resistant HGSOC and demonstrate that they preserve the original tumor architecture and key biomarkers (EpCAM, CA125, PAX8, HER2, MEK1/2, Cyclin E1), thus providing unique preclinical models for drug testing. These organoids were used to evaluate lurbinectedin in comparison with standard carboplatin and paclitaxel. While lurbinectedin showed comparable apoptotic effects to paclitaxel and superior activity to carboplatin, it induced chromosomal breaks to different extents in different cell lines, suggesting a distinct mechanism of action. Importantly, this work does not advocate for lurbinectedin as a superior therapy but, rather, demonstrates the utility of organoid models in uncovering drug-specific genomic effects. Our findings underscore the critical need for expanded testing using clinically relevant models to identify more effective strategies against PARPi-resistant disease.

## 1. Introduction

Epithelial ovarian cancer (EOC) is the fifth leading cause of cancer-related death in women and is the most lethal gynecologic malignancy in Western countries [1]. High-grade serous ovarian carcinoma (HGSOC) accounts for approximately 75% of EOC cases and is typically diagnosed at an advanced stage. First-line treatment consists of radical upfront or interval cytoreductive surgery and platinum-based chemotherapy with or without maintenance therapy consisting of the VEGF-inhibitor bevacizumab and/or a Poly(ADP-ribose) polymerase (PARP) inhibitor (PARPi), depending on the tumor’s molecular subtype. Although more than 80% of patients achieve initial remission, up to 90% experience recurrence within five years. Patients typically experience repeated relapses with progressively shorter remission periods, ultimately developing clinical platinum resistance and eventually succumbing to their disease [2].

PARP inhibitors have recently revolutionized the treatment of HGSOC. Approximately 50% of advanced HGSOCs exhibit homologous recombination deficiency (HRD), including pathogenic BRCA1/2 mutations or epigenetic silencing of HR-related genes, rendering these tumors highly susceptible to PARPi therapy [3,4]. PARP1 plays a critical role in the repair of single-strand breaks (SSBs) via the base excision repair (BER) pathway. PARP inhibition traps PARP1 on damaged DNA, processing unrepaired SSBs to double-strand breaks (DSBs) during DNA replication. In HR-deficient cells, particularly those with BRCA1/2 mutations, the inability to repair these DSBs via homologous recombination leads to synthetic lethality, resulting in catastrophic genomic instability and cell death [5,6]. Clinically, PARPi maintenance therapy following platinum-based chemotherapy has significantly improved outcomes in patients with HR-deficient tumors. The PAOLA-1/ENGOT-ov25 trial demonstrated a 5-year progression-free survival (PFS) rate of 66% in BRCA-mutated patients treated with olaparib, compared to 48% with placebo [2,7].

However, recurrence during or following PARPi maintenance is a common clinical event. As disease progression after PARP inhibition is associated with dismal outcomes—with a median PFS of just 6.6 months upon platinum reinduction—PARPi resistance represents a major clinical challenge [8]. Key resistance mechanisms include secondary *BRCA* reversion mutations, restoration of HR proficiency, and upregulation of drug efflux pumps [9,10]. The lack of effective therapeutic options for PARPi-resistant HGSOC underscores an urgent need for novel strategies.

Lurbinectedin, a synthetic marine alkaloid analogue, previously demonstrated clinical efficacy in recurrent HGSOC and may appear promising as a post-platinum therapeutic agent due to its platinum-like mechanism of action [11]. It binds to the central guanine of various nucleotide triplets in the minor groove of DNA and forms adducts inducing double-strand breaks, ultimately leading to apoptotic cell death [12]. Structurally similar to trabectedin, lurbinectedin’s modified tetrahydro-β-carboline group may enhance antitumor activity. Recent clinical evidence has indicated that trabectedin combined with pegylated liposomal doxorubicin demonstrates activity in PARPi-progressed disease [13], with expert consensus endorsing this combination as a third-line option [14]. However, clinical experience with trabectedin remains limited, and no data currently exist regarding lurbinectedin’s efficacy following PARPi progression, despite its compelling mechanistic rationale for this indication.

To our knowledge, no established preclinical models specifically for PARPi-resistant HGSOC have been published to date. Patient-derived organoids have recently emerged as powerful tools for solid tumor research [15,16], offering superior recapitulation of in vivo tumor architecture, functionality, and microenvironment compared to traditional 2D cultures. Additionally, they provide a more rapid, ethical, and cost-effective platform for drug screening than animal models. Thus, patient-derived organoids can be used to predict the responses of patients to treatments, therefore guiding therapeutic decisions [17,18].

Previously, we successfully established methods for cultivating HGSOC cell lines derived from patient materials and performed comprehensive molecular characterizations [19,20]. In this study, we established patient-derived cell lines from PARPi-resistant HGSOCs and subsequently generated the corresponding organoids from these cell lines. We analyzed the biological and molecular biological properties of these cell lines and organoids and compared them with the original tumor tissues. Furthermore, we evaluated the therapeutic potential of lurbinectedin, assessing its effects on the inhibition of cell proliferation and its cytotoxicity relative to carboplatin and paclitaxel.

## 2. Results

### 2.1. Clinical and Follow-Up Information of Patients and the Establishment of Patient-Derived Cell Lines

**Figure 1:**
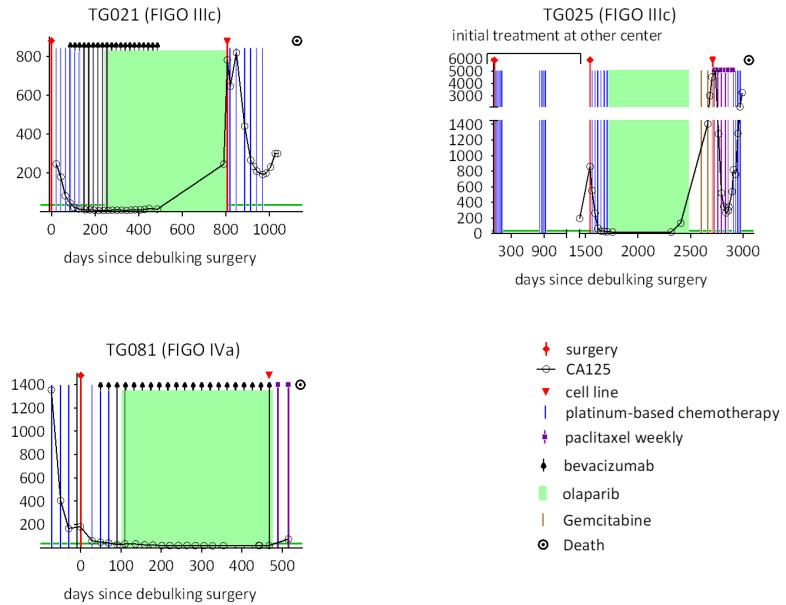
Treatment and clinical information for the donor patients are illustrated in individual graphs, each depicting the clinical course of one patient. Serial serum CA125 levels, serving as a surrogate for tumor burden, are plotted against days since the debulking surgery. Vertical bars indicate the timing and duration of chemotherapy regimens, while green blocks represent periods of ongoing PARPi treatment. Red triangles mark the time points at which cell lines were established. P36_TG021, P38_TG025, and P42_TG081 were treated with olaparib for 530, 700, and 357 days, respectively, and developed recurrent disease during treatment. They were then treated with carboplatin, gemcitabine/paclitaxel/carboplatin, and paclitaxel, respectively. None of these treatments led to remission, and the patients died during therapy.

Three patients with HGSOC who experienced disease progression following PARPi treatment were included in this study. Cell lines were established immediately after progression on PARPi therapy. The corresponding clinical data for these patients (P36_TG021, P38_TG025, and P42_TG081) are presented in Figure 1. Cell line establishment was performed as previously described [19,20]. While TG021 and TG081 were derived from ascitic fluid, TG025 was established from fresh tumor tissue.

### 2.2. Patient-Derived Cell Lines Present Varying Morphology and Proliferation Rates

**Figure 2:**
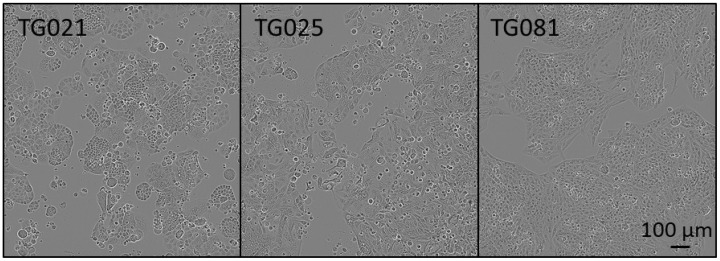
Morphology of the patient-derived cell lines.

The three cell lines displayed significant morphological variation. TG025 exhibited an elongated shape, whereas TG021 and TG081 appeared round. All lines proliferated as clusters (Figure 2). Cell diameters measured 12 μm (TG021), 13 μm (TG025), and 11 μm (TG081).

**Figure 3:**
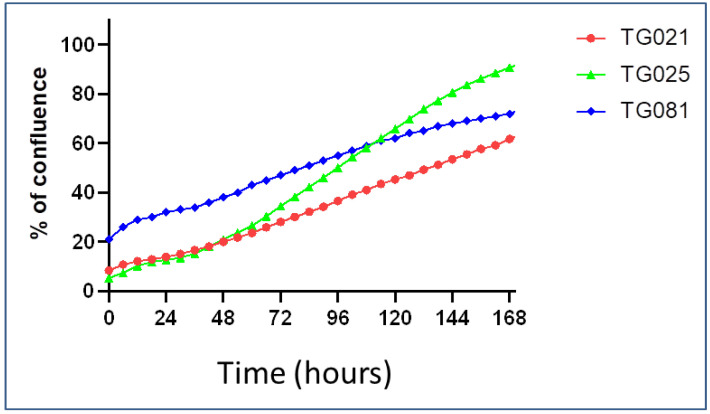
Growth curves of the cell lines. The average % confluence from triplicate measurements is plotted against time as lineal values.

Proliferation rates also differed markedly: TG021 and TG025 had doubling times of 48 h (2 days) and 36 h (1.5 days), respectively, while TG081 proliferated more slowly, requiring 108 h (4.5 days) to double (Figure 3).

### 2.3. Genetic Characterization of TP53, KRAS, and BRCA1/2

All three cell lines carried *TP53* point mutations resulting in amino acid changes (Table 1). In contrast, no *KRAS* mutations were detected. TG021 and TG025 additionally harbored *BRCA1* mutations, while *BRCA2* remained wild-type in both lines (Table 1). While no clinically significant *BRCA1/2* mutations were found in TG081, this cell line demonstrated homologous recombination deficiency, as assessed via Myriad myChoice® CDx PLUS assay.

### 2.4. Organoids from Patient-Derived Cell Lines Recapitulate Original Tumor Characteristics

**Figure 4:**
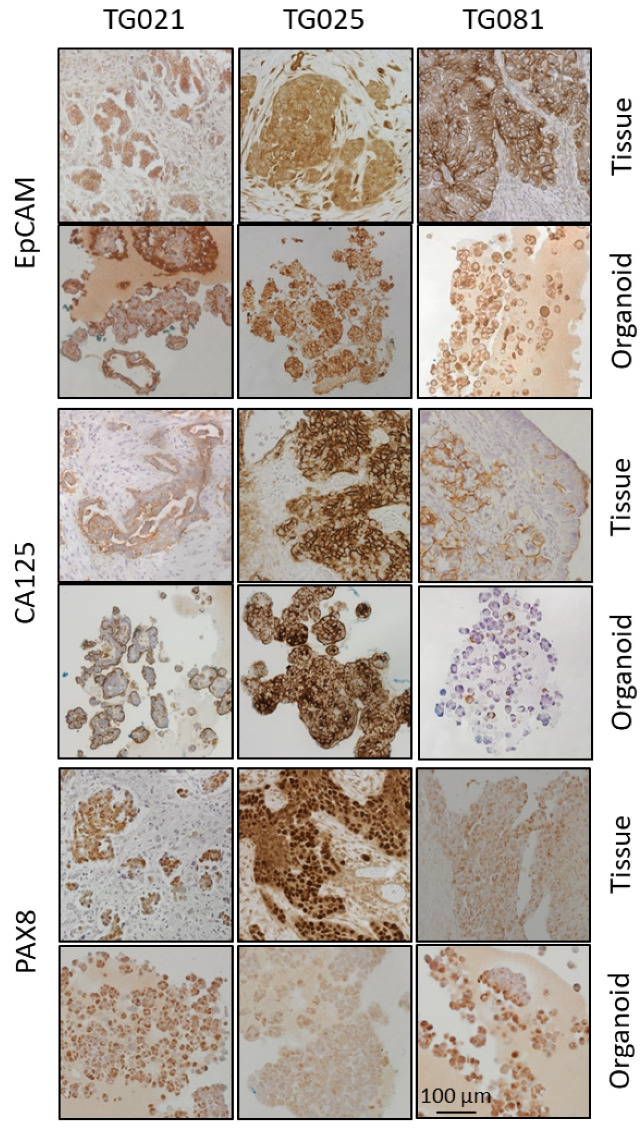
IHC staining results for EpCAM, CA125, and PAX8 in tumor tissues and their corresponding organoids.

All patient-derived cell lines successfully formed organoids in Matrigel, developing architectural features that resembled that of their parental tumors. Immunohistochemical analysis demonstrated conserved expression of tumor-specific markers between the original tumors and their corresponding organoids. As shown in Figure 4, all three lines expressed the characteristic HGSOC markers EpCAM, CA125, and PAX8. EpCAM, a membrane protein, was clearly detectable in both tumor tissues and organoids. CA125 expression varied across lines, with strong staining in TG025, weaker expression in TG021, and heterogeneous (partially positive) expression in TG081. PAX8, a nuclear marker indicative of HGSOC, was consistently expressed in all three lines and was maintained in both tissue and organoids.

**Figure 5:**
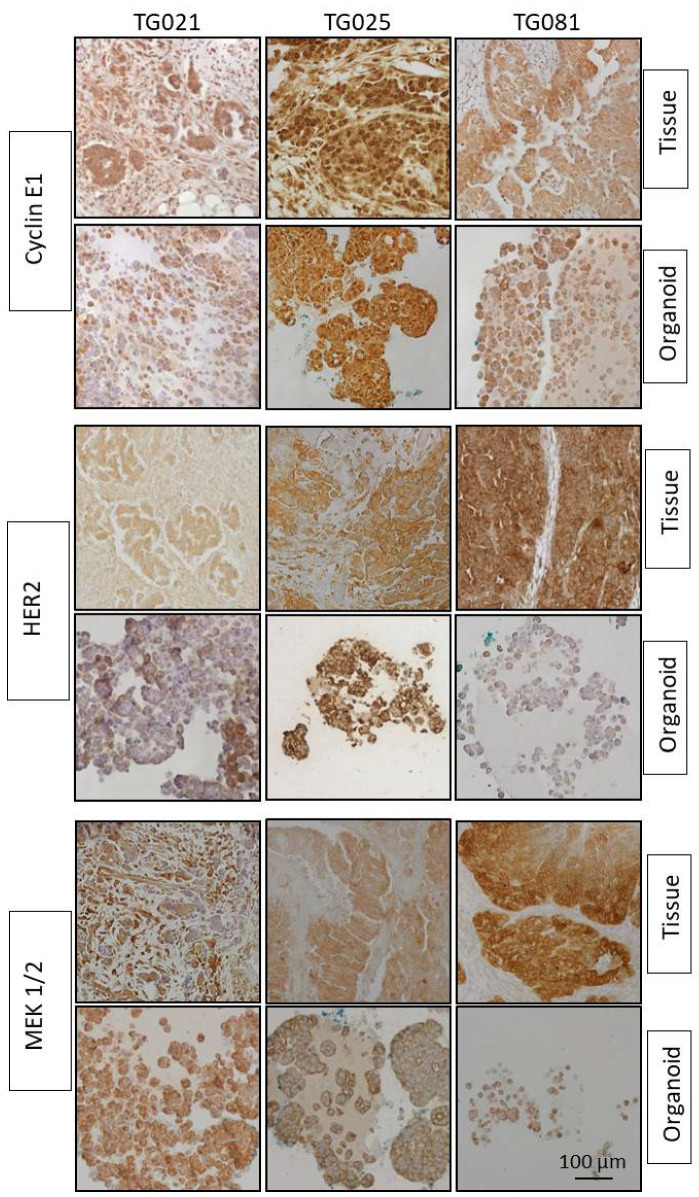
IHC staining results for therapeutic target markers Cyclin E1, HER2, and MEK 1/2 in organoids and their corresponding original tumor tissues.

Furthermore, the organoids exhibited comparable expression of clinically actionable targets, including HER2, MEK1/2, and Cyclin E1, for which targeted therapies exist (Figure 5).

### 2.5. Differential Drug Responses Across Cell Lines to Both Lurbinectedin and Paclitaxel

**Figure 6:**
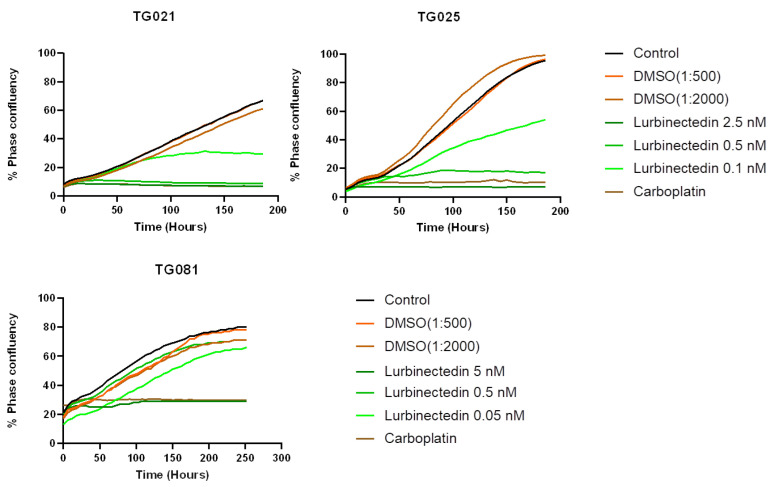
Response to lurbinectedin treatment. As TG081 previously showed similar responses to 0.1 nM, 0.5 nM, and 2.5 nM, a broader concentration range (0.05 nM, 0.5 nM, and 5 nM) was tested to better characterize dose-dependent effects. Only the test with the final concentration is presented.

The cell lines exhibited variable sensitivity to both lurbinectedin and paclitaxel. Live-cell imaging monitoring phase confluency revealed distinct responses to lurbinectedin treatment. At 0.5 nM concentration, lurbinectedin showed a minimal effect on TG081, while demonstrating efficacy comparable to carboplatin in TG021 and slightly reduced activity relative to carboplatin in TG025 (Figure 6).

**Figure 7:**
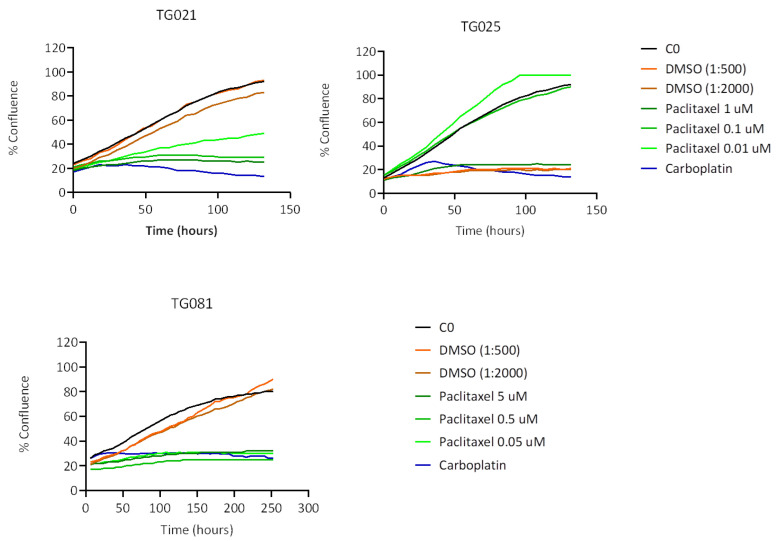
Response to paclitaxel treatment. Phase confluency is plotted against time (hours). DMSO was used as a control as paclitaxel was dissolved in DMSO.

In the case of paclitaxel, TG081 and TG025 demonstrated the highest sensitivity, exhibiting inhibition comparable to carboplatin at the lowest tested concentrations (0.05 μM and 0.01 μM, respectively). In contrast, TG021 showed only modest dose-dependent effects that did not exceed carboplatin’s efficacy (Figure 7).

**Figure 8:**
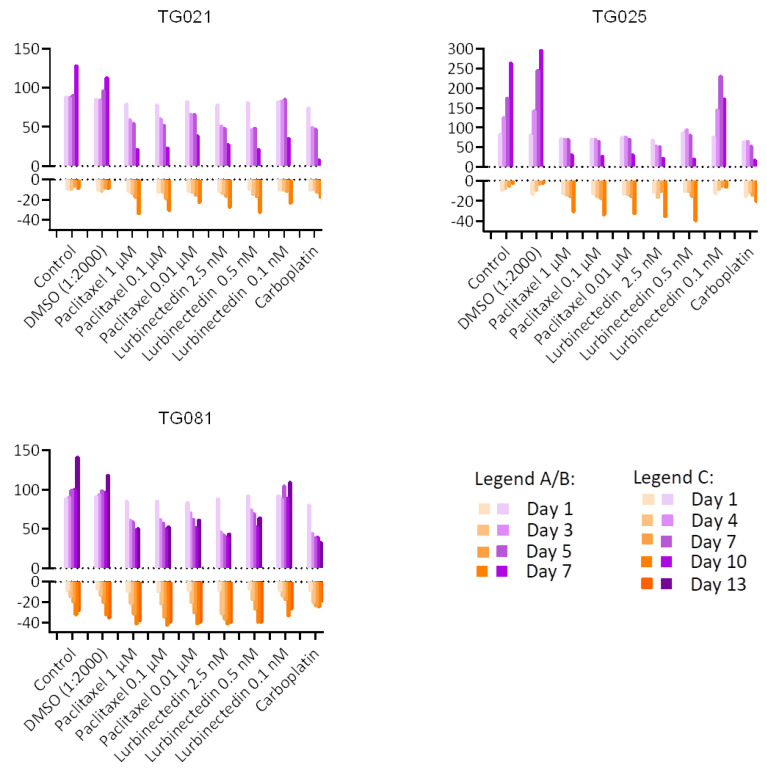
Comparative effects of lurbinectedin, paclitaxel, and carboplatin on cell proliferation and cytotoxicity. As TG081 had a slower proliferation rate than the other two cell lines, we performed the measurements at three-day intervals.

To evaluate the drugs’ effect on cell proliferation and cytotoxicity, we quantified live and dead cells separately. As shown in Figure 8, both lurbinectedin and paclitaxel exhibited marginally greater cytotoxic effects than carboplatin across all cell lines, though their anti-proliferative effects were comparable. The sole exception was TG025, where the lowest lurbinectedin concentration (0.05 nM) showed no significant effect on either proliferation or cell death (Figure 8).

### 2.6. Differential Genotoxic Effects of Anticancer Drugs on Chromosomes

**Figure 9:**
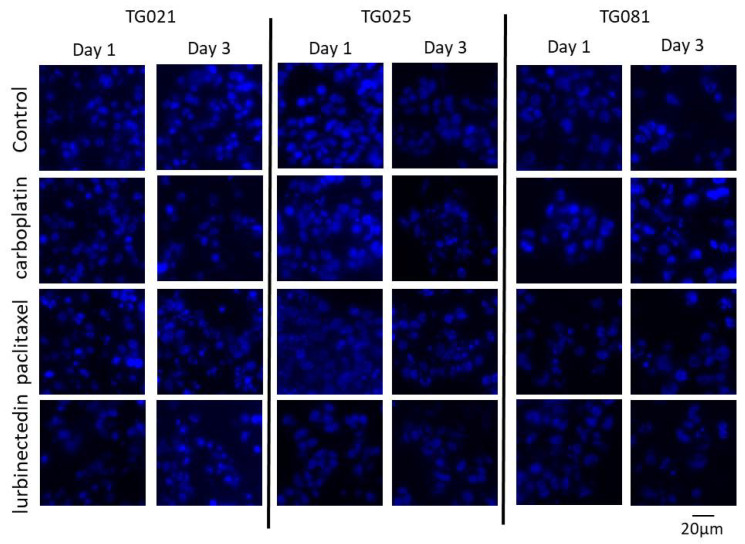
DAPI staining of drug-treated organoids. The organoids were treated with the corresponding drugs for 1 day and 3 days. They were harvested, fixed, embedded in paraffin, and sectioned for staining.

Chromosomal staining of organoids following 24- and 72-h drug exposures revealed distinct temporal patterns of genotoxicity. While carboplatin exhibited minimal effects on chromosomal integrity at 24 h, it had induced variable degrees of damage by 72 h. Paclitaxel demonstrated the most pronounced genotoxic effects and caused significant chromosomal breaks as early as 24 h, which further increased in severity by 72 h. Interestingly, lurbinectedin displayed an intermediate pattern: similar to carboplatin, it showed negligible effects at 24 h, but unlike carboplatin, it induced more consistent and measurable chromosomal damage across all cell lines by 72 h (Figure 9).

## 3. Discussion

In this study, organoids were successfully generated from patient-derived cell lines isolated from patients with PARPi-resistant HGSOC. Histopathological and immunohistochemical analyses confirmed that these organoids recapitulated the architectural and molecular features of the original tumors, including expression of established tumor markers (EpCAM, CA125, PAX8) and potential therapeutic targets (HER2, MEK1/2, Cyclin E1). These findings demonstrate that both the patient-derived cell lines and corresponding organoids constitute biologically relevant and valuable preclinical models for investigating the mechanisms of PARPi resistance in HGSOC.

Recurrent HGSOC poses significant treatment challenges due to its frequent development of resistance to PARP inhibitors, limiting therapeutic options and impairing patient outcomes. The lack of clinically relevant models for PARPi-resistant HGSOC has hindered progress in understanding resistance mechanisms and identifying effective alternatives. Therefore, there is an urgent need to explore two parallel strategies: (1) discovering novel molecular targets to overcome resistance, and (2) repurposing existing drugs with potential efficacy against these aggressive tumors. To address this gap, we established patient-derived cell lines and 3D organoid models from HGSOC tumors that recurred during PARPi therapy. These models retained the histological architecture and biomarker expression of the original tumors, mirroring their clinical behaviors. At present, they represent the only available systems that faithfully recapitulate clinically PARPi-resistant HGSOC, making them indispensable tools for molecular studies and preclinical drug screening. Leveraging these models, our work aims to accelerate the discovery of therapies tailored to resistant disease, ultimately improving survival for this under-served patient population. This study holds considerable importance for advancing personalized medicine, as we validated the utility of patient-derived cell lines and organoids for predicting drug efficiency. Furthermore, should a biobank of such models—annotated with comprehensive genetic, histopathological, and clinical data—be established, it could be used to tailor therapies for individual patients.

Patient-derived cancer cell lines grown in monolayer cultures often fail to fully recapitulate the structural and functional complexity of original tumors, due to the loss of critical microenvironmental interactions. However, when cultured under 3D conditions that mimic the in vivo niche, these cells can reorganize into organoids that closely resemble the native tumor architecture. This remarkable transition is fundamentally driven by the plasticity of cancer stem cells (CSCs), which possess the dual capacity for self-renewal and differentiation. CSCs can dynamically shift between epithelial, mesenchymal, and stem-like states in response to microenvironmental cues [21]; however, in 2D monolayers, mechanical constraints and lack of proper niche signaling suppress this plasticity, forcing cells into a homogeneous, undifferentiated state. The restoration of tumor architecture in 3D cultures occurs through two synergistic mechanisms. First, CSC plasticity is reactivated when cells are embedded in extracellular matrix (ECM)-rich environments (e.g., Matrigel or hydrogels containing laminin and collagen), allowing them to rebuild hierarchical tumor structures [22]. For example, in high-grade serous ovarian cancer (HGSOC), ALDH1+ or CD133+ CSCs regenerate glandular (EpCAM+/PAX8+) and invasive subpopulations, reconstructing the original tumor’s heterogeneity [16]. Second, soluble factors (e.g., Wnt, R-spondin) and ECM interactions reactivate developmental pathways (Notch, Hippo), reinstating the epigenetic and transcriptional programs of the parent tumor [23]. This is evidenced by HGSOC organoids re-establishing CA125 secretion and papillary structures that mirror the histology of primary tumors [24]. Thus, 3D organoids uniquely preserve CSC-driven plasticity and microenvironmental signaling, making them indispensable for modeling therapeutic resistance and tumor recurrence with higher fidelity than 2D cultures.

To explore alternative treatments for PARPi-resistant HGSOCs, we compared lurbinectedin with carboplatin and paclitaxel in terms of proliferation inhibition and cytotoxicity. Lurbinectedin showed comparable effects to paclitaxel but was less effective than carboplatin at inhibiting proliferation, despite demonstrating better cytotoxicity. These responses varied across cell lines, underscoring the heterogeneity of HGSOCs and the need for personalized approaches. Notably, lurbinectedin induced patient-specific chromosomal damage in organoids, unlike carboplatin or paclitaxel. This aligns with its reported mechanism of covalently binding DNA minor-groove residues, leading to S-phase delay, G2/M arrest, and cell death [25]. However, as lurbinectedin alone did not show significantly superior efficacy, it may not be ideal as a standalone therapy for PARPi-resistant HGSOCs. Lurbinectedin is structurally similar to trabectedin, which has demonstrated efficacy in PARPi-resistant disease. However, as the two compounds were evaluated in different experimental models, a direct comparison of their effects is not possible. Future studies using identical models would be valuable to compare their activities.

To assess the fidelity of our patient-derived organoids, we compared the expression and spatial distribution of key tumor markers (EpCAM, PAX8, and CA125) between organoids and their original tumor tissues. While the organoid lines TG021 and TG025 exhibited structures and staining patterns that largely matched those of their parental tumors, TG081 displayed notable deviations, including loose cell clusters. We noticed that the initial cell seeding density is a critical factor influencing organoid formation. Specifically, TG081 demonstrated slow growth kinetics, and substantial cell loss during trypsinization resulted in suboptimal seeding densities. This likely disrupted cell–cell contacts, impairing proper organoid assembly. To confirm this hypothesis, the experiment should be repeated with higher initial cell numbers to ensure adequate cell interactions.

Clinical records confirmed that all three patients developed resistance to both carboplatin and paclitaxel upon tumor recurrence during PARPi treatment, demonstrating acquired chemoresistance. While lurbinectedin has shown efficacy in platinum-resistant ovarian cancers [26], our functional assays revealed that this drug failed to outperform conventional chemotherapy in inhibiting cell proliferation. This lack of response likely stems from multiple factors, as PARPi-resistant subclones may harbor adaptive mechanisms (e.g., upregulated drug efflux pumps), their residual DNA repair capacity could mitigate lurbinectedin-induced damage, and protective tumor microenvironment signals may further promote their survival. Although lurbinectedin monotherapy did not demonstrated better effects for inhibiting cell proliferation when compared to carboplatin and paclitaxel, it demonstrated a stronger effect than carboplatin and an effect comparable to paclitaxel in inducing apoptotic cell death. These findings underscore the need to explore rational combination approaches and alternative targeting strategies in these aggressive tumors.

Drug resistance is not solely governed by the intrinsic molecular characteristics of tumor cells, but is also influenced by microenvironmental modulation and the roles of immune cells. To better understand these complex interactions, organoid models offer a valuable platform enabling the development of co-culture systems that capture the interplay between cancer cells and their surrounding environment. This approach provides a more physiologically relevant context for studying how stromal components, immune cells, and extracellular factors contribute to treatment resistance. Integrating tumor–microenvironment interactions into experimental models, patient-derived organoids pave the way for deeper insights into environmental influences on drug response, potentially uncovering novel therapeutic strategies to overcome resistance [27,28,29].

## 4. Materials and Methods

**Clinical Materials:** Tumor tissues or ascites from ovarian cancer patients were transferred from the Department of Obstetrics and Gynecology to the Department of Pathology at the Medical University of Vienna. Histopathological characteristics were independently assessed by two pathologists. The clinical specimens were then transferred to the laboratory for further processing. All procedures were approved by the local ethics committee (EK Nr. EK Nr. 1966/2020), and informed consent was obtained from all patients.

**Establishment of the cell lines and organoids:** Tumor tissue was scraped off in PBS using a cell scraper, and the resulting suspension was filtered through a 40 μm nylon cell strainer (Falcon, Corning Incorporated, Corning, NY, USA). Tumor cell clusters were washed with PBS and collected by inverting the cell strainer, followed by a final wash with medium. The cells were then seeded in either a 12.5 cm^2^ flask (Falcon) or a 25 cm^2^ Nunc EasyFlask (Nunc A/S, Thermo Fisher Scientific, Roskilde, Denmark) containing DMEM/F-12 (1:1) (1X) + GlutaMax-I medium (gibco, Life Technologies Limited, Paisley, UK) supplemented with 10% Fetal Bovine Serum (FBS), 100 Units/mL penicillin, and 100 μg/mL streptomycin (PS; all from gibco, Life Technologies Limited). Cultures were maintained at 37 °C in a 5% CO_2_ atmosphere. Ascites samples were filtered directly through a 40 μL cell strainer and processed identically. The initial cultures contained other non-tumor cells, which were depleted through repeated selective trypsinization using 0.05% trypsin (Gibco, Life Technologies Limited) for 2 min.

For organoid establishment from cell lines, approximately 5 × 105 cells were suspended in 10 μL of culture medium and gently mixed with 30 μL of Matrigel (Corning Matrigel Matrix, Corning Incorporated, Corning, NY, USA) on ice. The mixture was plated at the center of each well in either 4- or 24-well plates (Nunc A/S, Thermo Fisher Scientific). After incubation at 37 °C for 1 min to allow for partial gelation, plates were inverted and incubated for an additional 15 min at 37 °C. Organoids were subsequently cultured in the same medium described above.

**Live imaging for growth rate estimation and drug concentration validation:** The proliferation rate of each cell line was assessed using the IncuCyte SX3 live-cell analysis system (Satorius AG, Göttingen, Germany). Cells were seeded in 96-well Clear Flat Bottom plates (Corning® Incorporated) at an initial density of 20% and maintained at 37 °C with 5% CO_2_ for varying periods. Images were captured at 10× every six hours over the incubation period, with five fields of view captured per well. The average phase confluence percentage was plotted against time (hours) and the doubling time was estimated using the growth curve.

For drug testing, compounds at different concentrations were applied to cells seeded at an initial density of 10–20%. Cells were seeded in 200 μL medium and incubated overnight at 37 °C with 5% CO_2_. After incubation, the medium was replaced with 200 μL treatment solutions. Phase confluence percentage was plotted against time (hours). Carboplatin (10 mg/mL, Accord Healthcare B.V., Utrecht, The Netherlands), paclitaxel (5 mg, Invitrogen by Thermo Fisher Scientific, Hillsboro, OR, USA), and Lurbinectedin (1 mg, Hycultec GmbH, Beutelsbach, Germany) were aliquoted and stored according to the manufacturers’ instructions.

**Isolation of DNA and RNA:** For isolation of DNA and RNA from organoids, cells were first released from Matrigel. After washing twice with PBS, 1 mL of Corning® Cell Recovery Solution (Corning Incorporated, Bedford, MA, USA) was added to each organoid dome. The domes were then repeatedly pipetted up and down on ice for 5 min. Cells were collected by centrifugation at 400× *g* and 4 °C for 5 min, followed by two washes with 1 mL cold PBS. DNA and RNA were extracted using the AllPrep DNA/RNA Mini Kit (QIAGEN GmbH, Hilden, Germany), following the manufacturer’s protocol.

**Mutation detection of the*****TP53*****,*****KRAS*****, and*****BRCA1/2*****genes:***BRCA* mutational status was assessed as part of clinical routine using next-generation sequencing (NGS) on the Ion Torrent platform (Thermo Fisher Scientific, Waltham, MA, USA). Starting in 2020, tumor tissue was routinely subjected to homologous recombination deficiency (HRD) testing using the Myriad myChoice® CDx PLUS assay (Myriad Genetic Laboratories, Salt Lake City, UT, USA). Consequently, *BRCA* status is available for all cell lines, while HRD data are available for only one cell line. Mutations in *TP53*, *KRAS*, and *BRCA1/2* were re-assessed or confirmed from tissue of recurrent disease at the time of cell line establishment using the Ion AmpliSeq™ Cancer Hotspot Panel v2 (Thermo Fisher Scientific, Cat. No. 4475346).

**Preparation of Formalin-Fixed Paraffin-Embedded (FFPE) organoid samples:** Organoid domes were fixed in 4% paraformaldehyde (PFA) in PBS for one hour at room temperature, followed by two PBS washes and collection via brief centrifugation. Samples were then preserved in 70% ethanol at 4 °C overnight. For staining, organoids were treated with 1% alcian blue in 70% ethanol at 25 °C for 30 min. Subsequently, specimens underwent sequential dehydration steps: 96% ethanol for 10 min, 100% ethanol for 15 min, and xylene for 20 min, with each step repeated twice. Samples were then immersed in liquid paraffin for 30 min, followed by fresh paraffin replacement and an additional 30-min incubation on a warming plate. After hardening on a cooling plate for approximately 1 h, formalin-fixed, paraffin-embedded (FFPE) blocks were sectioned at 4.5 μm thickness using a microtome (HM 355 S, Thermo Fisher Scientific). Sections were mounted on slides, air-dried for 24–48 h at room temperature, and stored at −20 °C.

**Immunohistochemistry staining:** For immunohistochemical staining, slides were first incubated at 58 °C for 1 h and subsequently deparaffinized through two 5-min xylene washes. Rehydration was achieved by sequential 5-min incubations in 100%, 100%, 96%, 80%, and 70% ethanol. After thorough rinsing in distilled water, antigen retrieval was performed using either citrate buffer (pH 6.0) or Tris-EDTA (pH 9.0) that had been heated to boiling in a microwave and then allowed to cool to room temperature for 1–2 h.

Following drying, tissue sections were outlined using a hydrophobic barrier pen (Dako, Agilent, Santa Clara, CA, USA). Endogenous peroxidase activity was blocked with 3% hydrogen peroxide (H_2_O_2_) in methanol for 10 min at room temperature, followed by two distilled water washes. Non-specific binding was blocked using the ZytoChem Plus (HRP) Polymer Kit blocking solution (Zytomed Systems GmbH, Berlin, Germany) for 10 min, with subsequent washes in PBS-Tween. Primary antibodies, diluted in antibody diluent (Dako, Agilent) to the concentrations specified in Table 2, were applied at 100–150 μL per tissue section and incubated overnight at 4 °C.

The next day, slides underwent two PBS-Tween washes before incubation with the secondary antibody solution (Solution 2 from ZytoChem Plus kit) for 20 min. After additional PBS-Tween washes, the chromogen solution (Solution 3) was applied for 30 min at room temperature. For visualization, a substrate solution consisting of 1 mL substrate buffer mixed with 1 drop of DAB chromogen was applied (150 μL per slide) and developed for 2–15 min, with the development time adjusted based on color intensity. The reaction was stopped via two distilled water washes. Counterstaining was performed using hematoxylin (Merck KGaA, Sigma-Adrich, Darmstadt, Germany) for 1 s, followed by a 30–60 min rinse under running tap water. Finally, slides were preserved by mounting with Kaisers Glycerolgelatine (Merck KGaA, Sigma-Adrich).

**Cell Viability and Cytotoxicity Assessment:** To assess the relative number of live and dead cells, viability and cytotoxicity assays were performed using the CellTiter-Fluor™ Cell Viability Assay and CellTox™ Green Cytotoxicity Assay (both from Promega Corporation, Madison, WI, USA). Experiments were conducted in 384-well black-walled clear-bottom plates (Corning Incorporated, Kennebunk, ME, USA), with each well containing a total volume of 40 μL comprising equal parts cell suspension (20 μL) and treatment solution (20 μL). All assays were performed in DMEM/F-12, with no phenol red medium (Gibco, Life Technologies Limited). Cells were maintained under standard culture conditions at 37 °C in a humidified 5% CO_2_ atmosphere throughout the duration of the experiment.

Cell line-specific parameters were established as follows: TG021 and TG025 cell lines were seeded at 2000 cells per well with assessments conducted at 48-h intervals, while the TG081 cell line required higher seeding density (3800 cells/well) and was evaluated every 72 h due to its slower proliferation rate. The viability assay employed a fluorogenic peptide substrate (Gly-Phe-AFC) that detects live-cell protease activity, with reagent diluted 1:1000 in culture medium and incubated with cells for 30 min at 37 °C.

Cytotoxicity was assessed using the CellTox™ Green dye (1:1000 dilution), which exhibits enhanced fluorescence upon DNA binding in dead cells. Following incubation for 10 min at room temperature, fluorescence measurements for both assays were acquired using a CLARIOstar Plus microplate reader (BMG LABTECH GmbH, Ortenberg, Germany) with optimized settings: excitation/emission wavelengths of 380/505 nm for viability measurements and 485/520 nm for cytotoxicity detection. This dual-assay approach enabled simultaneous monitoring of treatment effects in both viable and dying cell populations within the same experimental system.

**Drug Testing in Organoids and DAPI Staining:** Drug treatments were administered to organoids following four days of initial cultivation, with subsequent molecular analyses performed at 24-h and 72-h time points post-treatment. Following drug exposure, organoids were processed for DNA/RNA isolation and prepared as FFPE blocks using the previously established protocol. For nuclear staining, FFPE sections were first incubated at 58 °C for 60 min, followed by deparaffinization through two sequential 5-min xylene washes. Tissue sections were then rehydrated using a graded ethanol series consisting of two washes with 100% ethanol for 5 min, 96% ethanol for 5 min, 80% ethanol for 5 min, and a final wash in 70% ethanol for 5 min. After thorough rinsing with distilled deionized water (ddH_2_O), sections were permeabilized with 0.5% Triton X-100 in PBS for 15 min at room temperature. Following permeabilization, slides underwent two 3-min washes in PBS and a final 2-min rinse in distilled water.

Nuclear staining was performed by incubating sections with DAPI (5 mg/mL stock solution diluted 1:100 in PBS) for 6 min at room temperature and protected from light. After staining, slides were washed twice with PBS containing 0.05% Tween-20 (PBS-T) for 3 min each, followed by a final rinse with Milli-Q® purified water (Merck Millipore, Darmstadt, Germany). Sections were mounted using Fluoromount-G™ aqueous mounting medium (SouthernBiotech, Birmingham, AL, USA) under light-protected conditions to preserve fluorescence signals. All stained slides were stored at 4 °C in the dark until microscopic analysis.

## 5. Conclusions

In conclusion, patient-derived organoids were successfully established from PARPi-resistant HGSOC cell lines, retaining the original tumor architecture and biomarker expression (EpCAM, CA125, PAX8, HER2, MEK1/2, Cyclin E1), thus validating their use as preclinical models. To identify alternative PARPi-resistant treatments, the efficacy of lurbinectedin was compared to that of carboplatin and paclitaxel. While lurbinectedin monotherapy showed no greater effect than carboplatin or paclitaxel in inhibiting cell proliferation, it exhibited stronger apoptotic activity than carboplatin and an effect comparable to paclitaxel. Notably, unlike carboplatin and paclitaxel, lurbinectedin induced heterogeneous chromosomal damage across tumor organoids, suggesting a distinct mechanism of action. These findings highlight the utility of organoid models for drug testing and highlight the need for more potent alternatives to standard therapies to treat PARPi-resistant HGSOC. The major limitations of the study are its small sample size, monotherapeutic testing, and the lack of consideration of microenvironmental factors.

## Figures and Tables

**Table 1:** Genetic information of the cell lines.

	*TP53*		*BRCA1*	
**Cell Line**	**Gene Mutation**	**Affected Protein**	**Gene Mutation**	**Affected Protein**
**TG021**	c.395A>G	p.(Lys132Arg)	c.2762del and c.2784_2803del	p.921_935QTVNITAGFPVVGQK>RQLISLQE *a
**TG025**	c.524G>A	p.(Arg175His)	c.3389_3390delAG and 3396delT	p.1267_1269EEN>GE *b
**TG081**	c.747G>T	p.(Arg249Ser)	wild-type	wild-type

Note: *a: c.2762delA caused a frame shift in the last codon of exon 10 and the subsequent 21 bases, resulting in 8 variant amino acids. The combined 1+20 bp deletion led to a 15 amino acid deletion and an 8 amino acid insertion. *b: c.3389-3390delAG and c.3396delT together caused a frame shift in two codons and a deletion of one amino acid, resulted in the amino acid sequence GE instead of EEN.

**Table 2:** Antibodies used for IHC.

Antibody	Company	Catalog Number	Dilution	Antigen Retrieval
EpCAM	Proteintech Europe, Manchester, UK	21050-1-AP	1:1000	Citrate
CA125	Leica Biosystems, Deer Park, IL, USA	NCL-L-CA125	1:200	Citrate
PAX8	Proteintech, Rosemont, IL, USA	10336-1-AP	1:750	Tris-EDTA
Cyclin E1	Abcam Limited, Cambridge, UK	ab71535	1:1000	Citrate
MEK1/2	Abcam Limited	ab178876	1:500	Tris-EDTA
HER2	Dako, Agilent	A0485	1:400	Citrate

## Data Availability

The original contributions presented in this study are included in the article. Further inquiries can be directed to the corresponding authors.
